# Using developmental evaluation to support knowledge translation: reflections from a large-scale quality improvement project in Indigenous primary healthcare

**DOI:** 10.1186/s12961-019-0474-6

**Published:** 2019-07-19

**Authors:** Alison Laycock, Jodie Bailie, Veronica Matthews, Ross Bailie

**Affiliations:** 1 Menzies School of Health Research, Charles Darwin University, PO Box 41096, Casuarina, Darwin, NT 0811 Australia; 2 The University of Sydney, University Centre for Rural Health, 61 Uralba Street, Lismore, NSW 2480 Australia

**Keywords:** Developmental evaluation, knowledge translation, dissemination, participatory research, Indigenous, quality improvement, stakeholder engagement, co-production

## Abstract

**Background:**

Developmental evaluation is a growing area of evaluation practice, advocated for informing the adaptive development of change initiatives in complex social environments. The utilisation focus, complexity perspective and systems thinking of developmental evaluation suggest suitability for evaluating knowledge translation initiatives in primary healthcare. However, there are few examples in the literature to guide its use in these contexts and in Indigenous settings. In this paper, we reflect on our experience of using developmental evaluation to implement a large-scale knowledge translation research project in Australian Aboriginal and Torres Strait Islander primary healthcare. Drawing on principles of knowledge translation and key features of developmental evaluation, we debate the key benefits and challenges of applying this approach to engage diverse stakeholders in using aggregated quality improvement data to identify and address persistent gaps in care delivery.

**Discussion:**

The developmental evaluation enabled the team to respond to stakeholder feedback and apply learning in real-time to successfully refine theory-informed research and engagement processes, tailor the presentation of findings to stakeholders and context, and support the project’s dissemination and knowledge co-production aim. It thereby contributed to the production of robust, useable research findings for informing policy and system change. The use of developmental evaluation appeared to positively influence stakeholders’ use of the project reports and their responses to the findings. Challenges included managing a high volume of evaluation data and multiple evaluation purposes, balancing facilitative sense-making processes and change with task-focused project management, and lack of experience in using this evaluation approach. Use of an embedded evaluator with facilitation skills and background knowledge of the project helped to overcome these challenges, as did similarities observed between features of developmental evaluation and continuous quality improvement.

**Conclusion:**

Our experience of developmental evaluation confirmed our expectations of the potential value of this approach for strengthening improvement interventions and implementation research, and particularly for adapting healthcare innovations in Indigenous settings. In our project, developmental evaluation successfully encompassed evaluation, project adaptation, capacity development and knowledge translation. Further work is warranted to apply this approach more widely to improve primary healthcare initiatives and outcomes, and to evaluate implementation research.

## Background

Developmental evaluation (DE) is a growing area of evaluation practice, developed to accommodate emergent programmes and projects. DE is used to inform adaptive development of change initiatives in complex environments [1–3]; however, there is limited literature describing its use in Australian Aboriginal and Torres Strait Islander (hereafter respectfully referred to as Indigenous) health programmes [4] or in knowledge translation research [5, 6]. This article is based on our experience of using DE to support the implementation of a theory-informed process defined as ‘interactive dissemination’. The process engaged stakeholders with aggregated continuous quality improvement (CQI) data from Australian Indigenous primary healthcare (PHC) services. We draw on knowledge translation principles and features of DE to reflect on the rationale, benefits and challenges of using DE in this large-scale project. We discuss the potential of DE for strengthening improvement interventions and for supporting knowledge translation and dissemination in PHC contexts.

### Indigenous people’s health and primary healthcare

Australia is a high-income country with large disparities in health outcomes between Indigenous and non-Indigenous people. The causes of this inequity include colonisation, land dispossession and associated trauma, socioeconomic inequality and racism [7]. Indigenous people access PHC through community-controlled and government-managed services established to meet their needs and through private general practices [8]. These PHC services are in diverse geographical settings and vary in size, resources and the range of services provided.

Improving health and well-being outcomes for Indigenous people in this complex healthcare environment requires change at multiple levels of the health system to support wide-scale improvement in the quality of PHC [9].

### Knowledge translation: theory-informed and interactive

Effective knowledge translation is important for closing the gaps between what we know and what is actually done in PHC [10]. It is critically important for addressing prevailing heath equity gaps between population groups, such as those that exist between Indigenous and non-Indigenous Australians [11]. Theory-informed knowledge translation and dissemination approaches are recommended when designing and evaluating interventions because they help to understand how knowledge is generated and used, to explain clinical and organisational behaviour, to inform strategy selection, and to understand effects [12, 13]. Much knowledge translation and dissemination literature describes the benefits of dialogue-based and interactive processes for moving research results into policy and practice [14–17]. In particular, participatory approaches that engage potential knowledge users as partners in solution- and impact-focused research are advocated [18, 19]. It is argued that bringing together users’ knowledge of the topic and implementation context with researchers’ expertise in methods and content results in relevant, actionable findings that are more likely to be used to improve care [20].

Consistent with these approaches, participatory action and partnership-based research are well established in CQI research in Australian Indigenous PHC [21, 22]. They have been used to co-develop evidence-based CQI tools and processes [23–28], to co-design and collaboratively conduct a large programme of system-based research [21, 22, 29], and to implement studies at the local level. These CQI research projects reflect understanding that successful improvement interventions in Indigenous contexts are those that incorporate Indigenous values and concepts of health and wellbeing [30, 31], draw on existing strengths, and are tailored to population health needs and to social, cultural, organisational and geographical settings [32–34].

### Developmental evaluation: utilisation and innovation focused

DE uses systems thinking to consider how multiple parts of complex and dynamic systems (such as healthcare systems) are interrelated, and focuses on users and real use of evaluation findings [35]. These features suggest suitability for evaluating projects that involve complex health system and translation issues, and which seek to engage multiple stakeholders in both research and change [36]. DE has been used to generate feedback as innovations are tested and to adapt programmes or products to their operating environments [37–39]. It has been used to modify products to suit new or changing contexts and users [37] and to engage communities of practice in systems change [1]. Other uses include strengthening the impact of multi-stakeholder research networks [40, 41] and developing collaborative processes between agencies addressing social challenges [42, 43]. DE positions evaluators as facilitators of change and embedded partners in innovation, and actively engages stakeholders in research, sense-making and change processes [1, 44]. These features support the utility of DE in strengthening participatory research processes and knowledge translation strategies and evaluating programmes in Indigenous settings, where DE has been used to develop or support innovative programmes that blend cultural and evaluation principles in contextually grounded approaches [4, 45, 46].

Our research team applied DE in a novel interactive dissemination strategy. The ‘Engaging Stakeholders in Identifying Priority Evidence–Practice Gaps and Strategies for Improvement in Primary Health Care’ (ESP) project (Box 1) engaged stakeholders in co-producing knowledge to inform system improvement for Indigenous health.

## Discussion

### Why use developmental evaluation in the ESP project?

The ESP project was novel in several respects – it was adapting knowledge translation theory [33, 47–49] to apply a CQI process at scale, using the largest available set of CQI data on Australian Indigenous PHC and it sought to engage people working in policy, management, CQI facilitator, health practitioner and research roles, in different geographical, organisational, social and cultural contexts, and at different levels of the health system in collective data interpretation and knowledge sharing. The ESP process aimed to draw on different types of knowledge (e.g. explicit, tacit, cultural) to identify common priorities, improvement barriers and enablers operating at individual, health centre/community and higher system levels, and possible ‘real-world’ solutions across the scope of clinical PHC. As would be expected, there was uncertainty about what processes, practices and products would work most effectively. Project implementation was certain to result in questions, challenges and successes that demanded real-time responses. We required an evaluation approach that could embrace this complexity and enable us to respond appropriately as needs and understandings evolved [1, 3, 37]. The approach also needed scope to appraise and adapt the theoretically informed research design [50].

Other factors favoured a DE approach. DE is characterised by repeated cycles of data collection, feedback, reflection and adaptation; the iterative research cycles of the ESP project were consistent with this feature of DE. Supporting innovators to bring about change that is tailored to group needs in complex, dynamic environments is a particular purpose of DE [35]. Our DE supported the engagement of stakeholders with CQI data to inform efforts to achieve multi-level system improvement in PHC systems for Indigenous people. Developmental evaluators are typically engaged as participant observers who guide data collection, inquiry and reflection-in-action [37, 51]. We had a team member who was able to undertake this role.

**Fig. 1:**
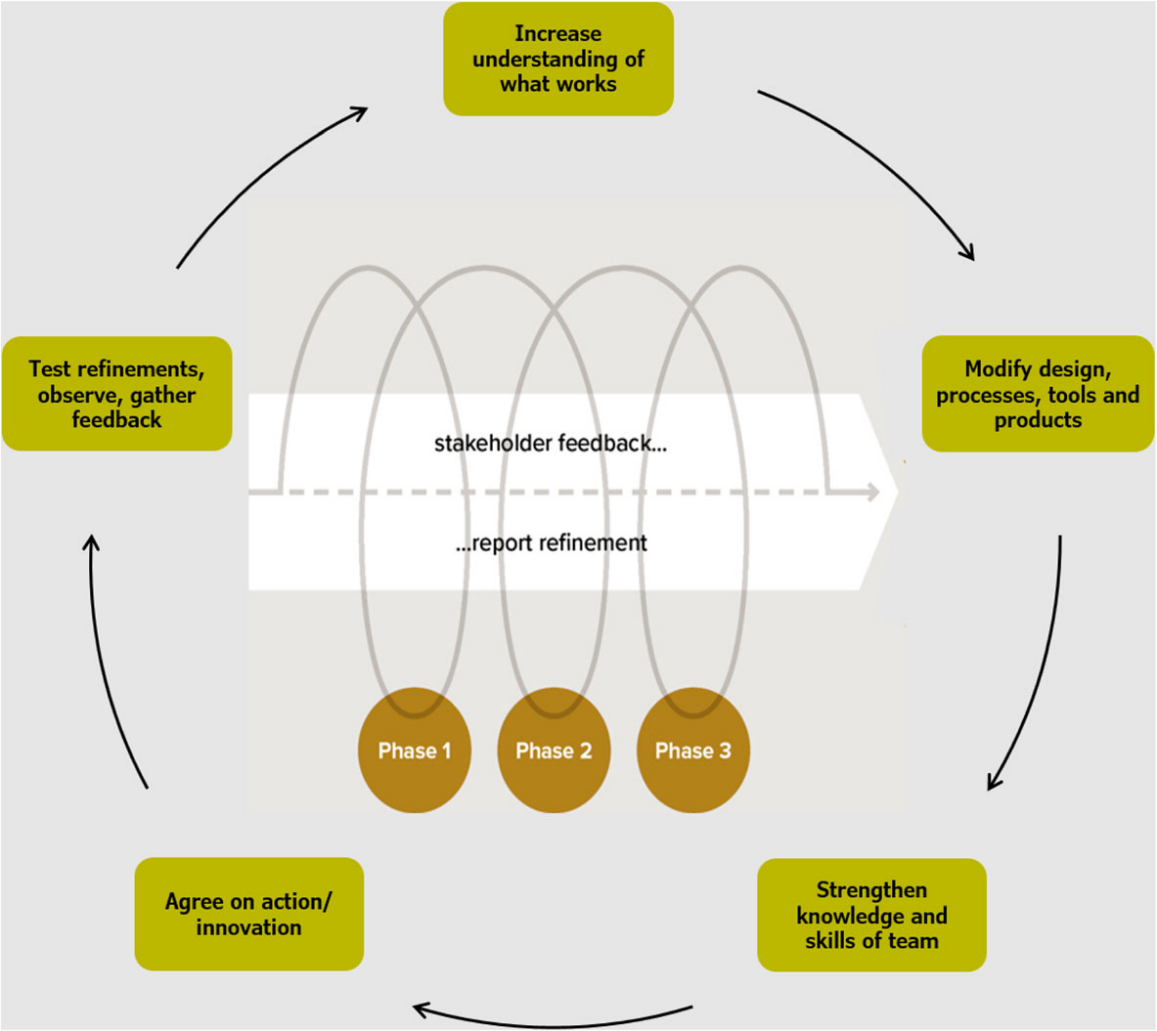
Systematically applying developmental evaluation in interactive dissemination cycles

The objectives of the DE were to (1) explore facilitators and barriers to stakeholder engagement with the data and use of ESP project findings; (2) inform ongoing project refinement and implementation and; (3) assess the utility of the interactive dissemination process [52]. Figure 1 illustrates how the DE was concurrently and systematically applied in the interactive dissemination cycles. The developmental evaluator drew on multiple sources of data, including project records, respondent surveys and semi-structured stakeholder interviews, as outlined in the study protocol [52]. These sources were used to facilitate reflective processes through which the team, which comprised one Indigenous and three non-Indigenous members, critically appraised ESP implementation and planned responses. Agreed refinements were tested, increasing our understanding of what worked (and did not work) and informing modifications to the project design, processes and reports.

### Benefits of using developmental evaluation

#### Continuous tailoring to strengthen stakeholder engagement and research outcomes

The DE as planned [52] provided specific effort and resources and enabled a systematic approach to the evaluation and refinement of the ESP process as it unfolded. It structured team time to regularly reflect on what occurred, analyse meaning and consider options for change. For example, a reflective workshop 3 months after project commencement was important for refining and consolidating ESP processes, team meetings were convened following rounds of stakeholder interviews, DE was a standing item in project administration meetings and discussions took place when evaluation data suggested changes were needed. Meetings of our wider CQI research network also provided opportunities to share evaluation findings with stakeholders, discuss project adjustments and generate further research translation ideas (e.g. visual representation of common findings across ESP cycles in different areas of care) [53].

Incorporating feedback from the target audience for the ESP reports led to tailoring and improvement in the process and the quality of reports and other communication resources. Changes could be tested and refined with each iteration of the ESP dissemination process.

These processes were important for supporting and maintaining stakeholder engagement. The target audience was widely dispersed across Australia and we were a small team using an online dissemination process. Evaluation cycles of data collection, reflection and change offset our limited interpersonal contact with stakeholders – they enabled us to demonstrate that we were responsive to feedback and to incorporate our growing understanding of the factors impacting on project participation and outcomes. We were also demonstrating a systematic process to continually improve ESP project implementation, in effect modelling CQI. This was perceived to strengthen the rigour and credibility of the research.

#### Knowledge contribution and knowledge sharing

The ESP project design was adapted from a systematic process developed by French et al. [48] to link interventions to modifiable barriers to address evidence–practice gaps. In order to capture stakeholder knowledge about barriers and enablers operating at health centre and wider system levels [33, 54], we made innovations to a questionnaire exploring individual attributes that influence care [47] based on the Theoretical Domains Framework [49]. DE enabled the team to continually appraise and refine these innovations, and to adjust the project design (e.g. by merging two reporting and feedback phases into one). As a result, the ESP process successfully engaged stakeholders in identifying priority evidence–practice gaps, improvement barriers, enablers and strategies at individual, health centre and system levels in each area of care. It captured responses from people representing a range of roles, organisations and healthcare contexts. Input from Indigenous people (e.g. Indigenous health service staff, members of governing boards of health services) ranged from 10% of survey respondents for the child health ESP to around 52% of survey respondents for maternal health [55]. DE helped us understand how and how well the theory-based interactive processes worked, and whether and how much the intervention processes could be adapted without compromising the research outcomes.

In addition, the large amount of data generated by the DE enabled us to apply a theoretical framework post hoc to assess the utility of the interactive dissemination process. The i-PARIHS framework was identified as a suitable analytical tool because it highlights ‘how’ implementation is activated with intended recipients in their contextual settings. It comprises four key constructs – facilitation, the innovation or evidence, recipients, and context [56]. Use of i-PARIHS as an analytical framework provided a deeper understanding of how well the ESP project worked (and did not work) to engage stakeholders in knowledge co-production. The DE process emerged as a facilitator of successful project implementation [55].

#### Real-time responses and applied learning

Positioning the evaluator within the team as a facilitator of dialogue and change supported timely responses. For example, when some people expressed uncertainty about whether the ESP surveys required their input (e.g. some clinicians thought the survey questions were more suited to policy-makers and vice versa), we modified communication templates. The modifications conveyed how input from different professional groups added value to the research and how the findings could benefit their work.

A key benefit of DE is its developmental function. Our DE findings could be applied in real-time to improve tailoring of the ESP project to stakeholders and context. What we learnt about engaging stakeholders with evidence, and about conducting participatory research at a systems level, was applied through actual changes to the research design, surveys, reports, communications and supporting resources [57] as the ESP project progressed. These changes could be appraised and refined through iterative DE processes. Examples of decisions and adaptations made in response to evaluation feedback are shown in Table 1.

**Table 1:** Examples of evaluation feedback, team decisions and adaptations

Evaluation findings	Team decisions and adaptations
Stakeholders in different roles had different information needs; some required summarised findings, others required detailed research reports	Report structure was adjusted to: 1-page key messages; 3-page executive summary; 25-page full report
The reports needed to be accessible and useful for a wide audience, including non-researchers; some stakeholders required more guidance to understand and use the reports	Report content was adjusted (e.g. to add an explanation of theory, to explain data trends, to add diagrams, to add a section on ‘how to use this report’)
Data tables were difficult for some stakeholders to understand and interpret	Presentation of health indicator and service delivery data was changed from table format to box-and-whisker-plot graphs^a^
Some stakeholders did not participate in the surveys because they lacked confidence in their data analysis skills	A link to an audio-visual resource was added to support the text explanation of how to interpret box-and-whisker-plot graphs^a^
Use of ‘academic-style’ language was a barrier to engaging with the reports	Plain language summaries were developed to accompany all subsequent reports
Some stakeholders did not participate in surveys because they perceived them to target those in other roles (e.g. policy officers perceiving the surveys targeted clinicians)	Statements on the advantages of participation by different professional groups were added to report summaries and emails
Some stakeholders found the surveys too long and/or considered the questions too repetitious	Survey questions were reduced in number across phase surveys; they were refined and reduced several times as the ESP project progressed
Many stakeholders who were motivated to participate had competing work demands and were time poor	Survey times were extended; email reminders were sent to encourage input
Those who participated in multiple ESP phases and cycles were committing considerable time; ‘survey fatigue’ was identified as a risk	Two project phases (one identifying barriers/enablers and one suggesting improvement strategies) were merged to reduce the number of phase surveys and reports in each ESP cycle
ESP emails were easily overlooked by key stakeholders due to high volumes of emails received	Coloured banners, photos and graphics were added to emails for more visual impact
ESP final reports were perceived to be large, partly because aggregated and trend data were included as appendices	Separate data supplements were published; they accompanied the ESP final reports
Expert input to data analysis and effective use of networks were important for stakeholder engagement	An expert/lead clinician in each area of care was asked to assist with data analysis, co-author ESP reports and disseminate the reports through their professional networks
CQI facilitators were vital for promoting the ESP project and facilitating local engagement	The team encouraged project communications and report dissemination through CQI practitioners/leaders in the CQI network
Indigenous stakeholders were mainly participating through group (rather than individual) survey responses	Group input was encouraged through project communications, in recognition of the critical importance of Indigenous input
A resource was needed to encourage and support group discussion and interpretation	A group facilitation guide was developed and promoted; links to the guide were embedded in report summaries and emails
One-page overviews of key findings in each area of care were suggested as a way of encouraging stakeholders to engage with the findings and access the ESP final reports	One-page overviews of key findings were produced and distributed
Key messages for action were needed to promote the use of ESP findings in each area of care	Key messages for action were developed from ESP findings; they were included in ESP reports and published as plain language summaries
Findings needed to be presented in a variety of formats to suit different work needs and learning styles	Findings were published online in all developed formats – reports, summaries, PowerPoint presentations, journal articles
ESP findings needed to be widely and easily accessible in the longer term; stakeholders intended to use the reports to resource future work tasks and information needs	ESP findings were published on research institution websites and in open web-based repositories for Indigenous health and policy publications

^a^ Box-and-whisker-plot graphs display the distribution of data based on the five-number summary: minimum, first quartile, median, third quartile, and maximum

*CQI* Continuous quality improvement, *ESP* Engaging Stakeholders in Identifying Priority Evidence–Practice Gaps and Strategies for Improvement in Primary Health Care

### Developmental evaluation challenges

#### Managing complexity and uncertainty

The characteristics of the ESP project that suited a DE approach – the novel use of aggregated CQI data, a previously untested dissemination process, complex PHC environment and a diverse target audience – sometimes resulted in ambiguous findings and uncertainty about the best way forward. It took time to appreciate that such uncertainty was typical in undertaking DE and to be comfortable with sense- and decision-making processes that occurred opportunistically.

We needed to be flexible and respond strategically to what was unfolding. This sometimes required us to revise previous decisions in the light of emerging patterns in feedback. For example, we initially dismissed the idea of merging separate surveys identifying barriers/enablers and strategies to maintain fidelity to the model on which the research was based. This decision was revised when competing work demands and lack of time were persistently identified as barriers to engaging with ESP reports and completing the surveys. Following the change, we monitored the quality of survey data and added an evaluation question inviting feedback about the change.

#### Using an embedded evaluator

Team members had experience with traditional evaluation approaches that position the evaluator externally to ensure independence and objectivity. An evaluator who was embedded in the team as a participant observer, with in-depth knowledge of the project background and context, challenged this principle. However, we found that background knowledge supported more nuanced understanding of what was occurring in the ESP project and facilitated real-time tailoring to Indigenous PHC stakeholders (e.g. providing a group facilitation guide and working with CQI network members to encourage input that reflected cultural, community and service perspectives). Reflexive practice [58] reduced the risk of making assumptions about stakeholder needs. The evaluator was based in a different physical location to the team and this provided some independence from day-to-day project operations.

An embedded evaluator blurred role boundaries. As innovators we all became evaluators [3] and the evaluator was responsible for implementing some innovations (e.g. writing plain language summaries of ESP reports). Our prior experience in action research may have helped to prevent potential role tension. Rey et al. [5] liken a DE approach to conducting action research, explaining that DE evaluators engage in experiential learning cycles to both produce knowledge and facilitate change. In spanning the boundary between researchers and stakeholders, our evaluator helped to achieve the project’s knowledge co-production aim. There are examples of this boundary-spanning role being undertaken by academic researchers who are embedded in host organisations for knowledge co-production projects [59, 60]. In addition, prior working relationships and complementary skill sets were facilitative factors in managing these interactions and making best use of time and skills across the team.

#### Committing sufficient resources and managing data

Responding to continual processes of reflection and inquiry impacted on the project timeframe and resource needs. It required a range of skills and team capacity (e.g. to change data visualisation, to work flexibly with experts who assisted with analysis). Having a team leader committed to DE as well as an experienced team member who wanted to do post-graduate study in this topic area enabled us to surmount the normally significant challenge of resourcing a DE over an extended period. The high level of benefit delivered by these circumstances may not be available to teams with fewer resources and tight timeframes.

Managing high volumes of evaluation data and distinguishing between evaluation data and ESP project data were ongoing DE challenges. Collecting and synthesising large amounts of data in a timely way is an identified challenge of DE [37, 61], and our evaluation aimed to build a contextualised and integrated understanding of the findings and evaluation outcomes of a data-driven research project [52]. To achieve this, the DE drew on ESP project documentation and data, reviewed online survey data and ESP reports, monitored project adaptations, and collected and analysed data obtained through stakeholder interviews. This occurred concurrently with the ESP team’s production of 18 research reports and stakeholder surveys, 6 data supplements and other knowledge translation products. The evaluator had a direct role in some of these project tasks. Balancing DE processes with task- and results-focused ESP project management demanded decisive project leadership, good planning and teamwork, and flexible DE processes.

#### Lack of experience with this evaluation approach

No members of the team had previously participated in a DE, including the evaluator. The uncertainty inherent in DE, and the paucity of literature describing methods used in DE, caused the evaluator to regularly reflect on whether our evaluation was indeed developmental. Patton recently identified eight principles that should be addressed within a developmental evaluation [1, 35]. To assist research teams considering the use of DE, we describe ways in which we now understand the evaluation of the ESP project to reflect these principles (Box 2).

### Developmental evaluation and continuous quality improvement

We found DE to be congruent with the way we work in CQI. Evaluation literature identifies the purpose of DE as responding to changes (e.g. in understanding, participants or context) by doing something differently. Patton contrasts this with the improvement purpose of many formative evaluations [62], a comparison that suggests DE might be challenging for researchers coming from a quality improvement perspective. However, adapting the ESP project to improve the relevance and use of data and ESP reports among stakeholders seemed consistent with the DE purpose. Furthermore, both DE and CQI can involve complexity and systems thinking. Both approaches feature client-focused, participatory processes and both involve iterative data-informed reflection, decision-making and change.

Applying DE processes in the ESP project could be likened to using ‘plan-do-study-act’ cycles. We collected and interpreted data, worked out change strategies, implemented them, evaluated how they worked and repeated the cycle with different sets of PHC CQI data. DE processes also encouraged us to draw on CQI theory and practice as well as our experience of participatory research to think more deeply about the role of facilitation in the ESP project [55].

The ESP cycles could themselves be likened to scaled-up ‘plan-do-study-act’ cycles. ESP reports presented health centre performance data, which were used to identify improvement priorities and strategies that took account of contextual and workforce factors. However, applying these processes at scale to focus on system-wide improvement was new and involved complex interactions, making it difficult to predict the adaptations required to support engagement and ensure robust research findings. DE adequately addressed this challenge [37, 38].

### Using developmental evaluation to advance knowledge translation

We observed that DE acted as a knowledge translation process. Firstly, the successful implementation of knowledge co-production at scale without intense facilitation effort appeared partly due to the facilitative function of DE. By supporting continuous adaptation and tailoring to stakeholders and context, DE helped to identify and foster the ESP facilitation efforts of key stakeholders and CQI champions in workplaces [55].

Secondly, participation in the surveys and evaluation interviews [52] prompted stakeholders to think more deeply about how the reports could or should be used for Indigenous health improvement. Evaluation data show that it stimulated them to use the reports in a variety of ways [63], and to pass project information on to others and encourage others to use the reports or respond to the key findings. As a result, stakeholders working at different system levels used the ESP reports for complementary purposes (e.g. reflecting on individual practice, building team skills in data analysis, programme planning, influencing policy, developing new research) [63]. A multi-level improvement approach has greater likelihood of achieving change [9]. An evaluation approach with the ability to strengthen evidence use at multiple levels potentially has a role in creating synergy for improvement.

Third, evaluation feedback provided guidance on encouraging people to engage with and discuss the data, reflect on practice, community and system contexts, and share perspectives on improving care. This included guidance to increase Indigenous stakeholder input into data interpretation (e.g. by providing resources to support the participatory interpretation of data). Stakeholders reported learning new skills in data analysis and being stimulated by the improvement ideas of others [55, 63]. CQI research in Indigenous PHC indicates that support for pooled knowledge assists engagement in improvement initiatives [21, 64, 65]. It is also recognised that co-production can have subtle impact on research capacity-building and knowledge sharing, as well as demonstrable benefits such as policy and practice [66]. The DE supported knowledge pooling and co-production (e.g. by informing survey refinement and encouraging group input). At a higher level, using a developmental approach informed adaptation of the theory-based processes used in the ESP project and, ultimately, our positive assessment of the utility of the interactive dissemination process [55].

Finally, the concurrent ESP and DE processes assisted in maintaining stakeholder engagement through iterative ESP cycles to identify common evidence–practice gaps and common perceptions of the enablers and barriers to addressing those gaps across different areas of care (e.g. child health, chronic illness) [67].

While recognising that improvement strategies need to take account of local context, the common findings can be used to target policies and system interventions to improve health service performance and Indigenous health outcomes.

### Developmental evaluation limitations and future research priorities

The intended purposes of our evaluation went beyond adapting an innovative knowledge translation process. We needed to make judgements relating to its merit and utility (which is more aligned with summative evaluation than developmental purpose), and to generate knowledge to inform future translation initiatives and evaluations [52]. In reality, we needed to apply a combination of evaluation approaches, including the use of an analytical framework to assess the success of project implementation.

Further to the challenge we experienced in defining the boundaries between the ESP project work and the DE, delineating ESP project-related data and the DE data (e.g. in survey feedback) was often difficult. Despite flexible timeframes, data-related tensions regarding time and budget constraints emerged. For example, taking sufficient time to synthesise, reflect on and respond to evaluation findings was integral to the DE and important for maintaining stakeholder engagement. Conversely, project momentum was important because the ESP reports were valued as a source of robust PHC data available in real time. It was not within the scope of the project for the DE to adapt reports and processes for individual settings (e.g. PHC centres) and appropriate responses to DE data were not always feasible (e.g. the team was not resourced to facilitate groups for data interpretation, as consistently recommended by stakeholders). However, not acting on feedback risked disengagement by stakeholders. In addition, stakeholders had differing perspectives about the project changes needed. DE processes identified, but could not necessarily resolve, these tensions.

Future research could explore the use of DE to strengthen knowledge translation processes and to support Indigenous engagement in bringing about change. The use of DE to support interactive dissemination processes could be extended to engage PHC clients/consumers with CQI data for decision-making about health and context-specific improvement interventions.

Use of DE when applying interactive dissemination processes in other health settings would further the understanding of the elements and resources needed for successful knowledge co-production. DE should be further explored as a method for informing the scale-up of participatory research and improvement interventions and as an alternative to the more traditional process evaluation approaches adopted in implementation and improvement research.

## Conclusion

Our experience of DE confirmed our expectations of the potential value of this type of work for strengthening improvement interventions and knowledge translation research. In the ESP project, DE encompassed project implementation, evaluation, capacity development and knowledge translation. It supported the use of implementation theory to enhance the development and evaluation of our improvement research. While every situation and group will be different, the benefits of applying DE attest to its suitability for adapting and evaluating PHC innovations in Indigenous settings. Lessons learnt have enhanced our skills and knowledge about what works to engage Indigenous PHC stakeholders with data for knowledge co-production and system-wide change and, more generally, how to add impact and value to CQI research through research translation. Available resources, including facilitation skills and time, and scope for flexibility and change within a project or programme will influence the feasibility and benefits for teams adopting this evaluation approach. Further research is warranted to advance knowledge about the effective use of DE to improve translation and healthcare initiatives and outcomes.

Box 1 The Engaging Stakeholders in Identifying Priority Evidence–Practice Gaps and Strategies for Improvement in Primary Health Care (ESP) project (2013–2016) and developmental evaluation The ESP project [50] was a Flagship Project of the National Health and Medical Research Council-funded Centre for Research Excellence in Integrated Quality Improvement (CRE-IQI) [41]. It built on the Audit and Best Practice in Chronic Disease (ABCD) programme of continuous quality improvement (CQI) action research, which employed a systems approach to improving care delivered through Indigenous PHC services across Australia [21, 22, 68]. Agreements between health services and the ABCD National Research Partnership [22] enabled CQI data to be gathered over a decade from 175 Indigenous primary healthcare (PHC) centres spread across five Australian jurisdictions (38 Indigenous community-controlled and 137 government-managed health services).

The ESP project aimed to (1) disseminate the regionally and nationally aggregated CQI data on health systems performance in different aspects of PHC and (2) engage PHC stakeholders working in diverse roles, organisations and contexts in using the data to identify priority evidence–practice gaps, barriers and enablers, and to suggest strategies for improving care. An interactive dissemination process used phases of data reporting and stakeholder surveys to co-produce this knowledge, culminating in final ESP reports.

*Phase 1: Identification of priority evidence–practice gaps.* A preliminary analysis report of aggregated cross-sectional CQI data was distributed with a linked online survey.

*Phase 2: Identification of barriers, enablers and strategies for addressing identified gaps in care.* A report of trend data relevant to the identified priority evidence–practice gaps was distributed. Respondents completed an online survey about influences on individual behaviours, the health centre and wider systems and strategies they would suggest for modifying barriers and strengthening enablers.

*Phase 3: Provision of feedback on draft final report*. The report was distributed with an online survey gathering feedback on the draft overall findings.

The process was repeated using aggregated CQI data for child heath, chronic illness care, maternal, preventive and mental health, and rheumatic heart disease care [50].

Within these repeated cycles, developmental evaluation data were collected using mixed methods:

*Document review*: Project records of team communications and meetings, key project decisions and adaptations, ESP reports and processes, feedback gathered at twice-yearly meetings of CRE-IQI network members and other forums.

*Stakeholder surveys*: Each phase survey (above) included evaluative questions about the ESP reports and processes using Likert-type scale and/or text comments.

*Stakeholder interviews*: Interviewees represented a range of PHC roles, organisation types and settings, and participation in ESP processes in different areas of care (e.g. child health, mental health).

*Team reflection*: Facilitated reflection processes were ongoing throughout the project [52].

Box 2 How well did the evaluation of the ESP project reflect developmental evaluation principles?

1. ***Developmental purpose*:** Our evaluation aimed to inform and support the ongoing development of a novel dissemination strategy.
2. ***Evaluation rigour*:** Specific effort and resources and a systematic approach were used to gather, analyse and interpret evaluation data. Change decisions were evidence-based, involved the team in critical and creative thinking, and were appraised through ongoing evaluation cycles. They were recorded in DE monitoring logs and tracked through project refinements.
3. ***Utilisation focus*:** Our evaluation focused on intended use by intended users from start to finish in two respects – (1) the team’s use of evaluation data to strengthen the interactive dissemination process and (2) stakeholders’ use of CQI data to identify priority evidence–practice gaps, barriers and strategies for improving care.
4. ***Innovation niche*:** Innovative aspects of the project included the scale of the participatory CQI process, the use of aggregated CQI data, open online recruitment of participants and knowledge sharing by people at different system levels. Innovations to an existing implementation tool [47, 49] were made and tested, with potential for use in other contexts.
5. ***Complexity perspective*:** Recognising the complexity of the Indigenous PHC environment, the evaluation aimed to identify, understand and address factors that influenced stakeholder engagement in the project and their use of findings (e.g. by improving presentation of data, by supporting team discussions about improving care with customised resources, by including different messages for different target groups).
6. ***Systems thinking*:** The ESP dissemination process was designed to cross professional, organisational and geographical boundaries and capture different perspectives in data interpretation. Consistent with ESP project aims, the evaluation sought input from people in different settings and in policy, management, health practitioner, CQI and research roles. It explored how interactions between the team and stakeholders, and between stakeholder groups, impacted on project implementation, using the knowledge to refine the project. ESP processes successfully identified evidence–practice gaps and barriers that occurred system wide, providing evidence and opportunities for higher-level system change.
7. ***Co-creation*:** The ESP project and the evaluation were developed and refined together (e.g. Survey questions: in early ESP phases and cycles, Likert-scaled items were used to assess the acceptability and usefulness of ESP reports and surveys; once formats and processes were established, we invited comments about knowledge-sharing processes, learning and ways to improve the project). The co-creation principle was reflected in the ESP project aim, which was for researchers and PHC stakeholders to co-create knowledge for improving PHC and the project processes. The team acquired knowledge and skills in research translation through interactions with stakeholders, and lessons learnt were shared through ESP reports and processes. The co-creation principle aligns with our ‘All teach, All learn’ research capacity strengthening approach in Indigenous PHC CQI [69].
8. ***Timely feedback*:** Evaluative questions were included in ESP phase surveys from the outset and influenced early change decisions. Evaluation interviews were spread across different ESP phases and data cycles. Continuous feedback and an embedded evaluator enabled change decisions to be made and enacted in real-time.

*CQI* Continuous quality improvement, *DE* Developmental evaluation, *ESP* Engaging Stakeholders in Identifying Priority Evidence–Practice Gaps and Strategies for Improvement in Primary Health Care.

## Data Availability

Not applicable.

## Abbreviations

CQI: continuous quality improvement
DE: developmental evaluation
ESP: Engaging Stakeholders in Identifying Priority Evidence–Practice Gaps and Strategies for Improvement in Primary Health Care
PHC: primary healthcare
